# NCOA4-Mediated Ferritinophagy: A Potential Link to Neurodegeneration

**DOI:** 10.3389/fnins.2019.00238

**Published:** 2019-03-14

**Authors:** Maria Quiles del Rey, Joseph D. Mancias

**Affiliations:** Division of Genomic Stability and DNA Repair, Department of Radiation Oncology, Dana-Farber Cancer Institute, Boston, MA, United States

**Keywords:** NCOA4, ferritinophagy, iron homeostasis, ferroptosis, neurodegenerative disorders, neurodegenerative disease, neurodegeneration

## Abstract

NCOA4 (Nuclear receptor coactivator 4) mediates the selective autophagic degradation of ferritin, the cellular cytosolic iron storage complex, thereby playing a critical role in intracellular and systemic iron homeostasis. Disruptions in iron homeostasis and autophagy are observed in several neurodegenerative disorders raising the possibility that NCOA4-mediated ferritinophagy links these two observations and may underlie, in part, the pathophysiology of neurodegeneration. Here, we review the available evidence detailing the molecular mechanisms of NCOA4-mediated ferritinophagy and recent studies examining its role in systemic iron homeostasis and erythropoiesis. We propose additional studies to examine the potential role of NCOA4 in the brain in the context of neurodegenerative diseases.

## Introduction

Iron is an indispensable element for nearly all living organisms as it takes part in a wide range of biological reactions such as oxygen transport, oxidative phosphorylation, and enzymatic reactions required for cellular proliferation (Pantopoulos et al., 2012; Bogdan et al., 2016; Muckenthaler et al., 2017). While iron is critical for these reactions, iron availability must be tightly regulated as high levels of free iron can generate deleterious reactive oxygen species via the Haber-Weiss/Fenton reactions (Dixon and Stockwell, 2014). Many neurodegenerative disorders (ND), including Alzheimer’s Disease (AD), Parkinson’s Disease (PD), Huntington’s Disease (HD), and Amyotrophic Lateral Sclerosis (ALS) are associated with elevated free iron, dysfunctional iron homeostasis in general, and resultant oxidative stress that can precipitate ferroptosis, an iron-dependent form of cell death (Ward et al., 2014; Kim et al., 2015). Likewise, a group of inherited neurological disorders known as Neurodegeneration with Brain Iron Accumulation (NBIA) are characterized by inappropriate iron deposits associated with oxidative stress and neuronal cell death (Kruer, 2013).

**FIGURE 1:**
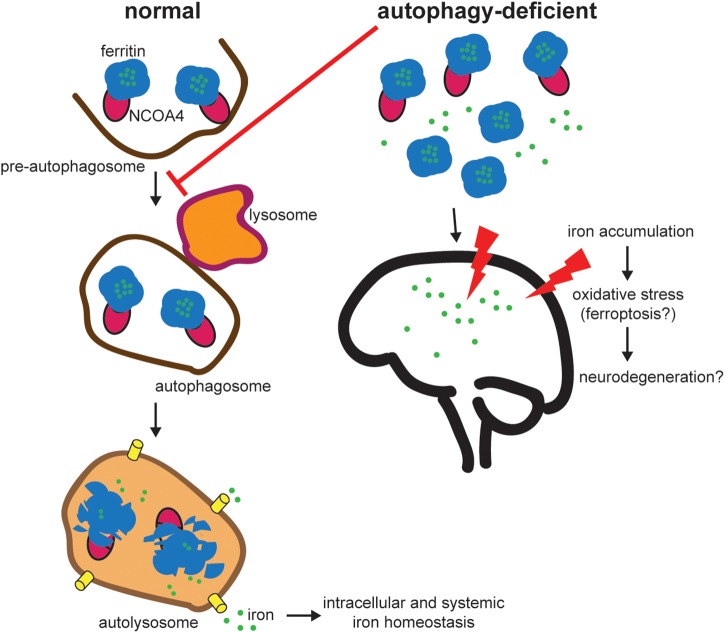
NCOA4-mediated ferritinophagy modulates intracellular and systemic iron homeostasis. NCOA4 binds iron-laden ferritin and delivers it to a pre-autophagosome structure. Upon autophagosome-lysosome fusion, ferritin is degraded, iron is released into the lysosome and exported to the cytosol for use in multiple iron-dependent physiological processes (e.g., mitochondrial heme synthesis). Under pathologic autophagy-deficient conditions, one model predicts that NCOA4 is unable to target ferritin for lysosome degradation leading to accumulation of ferritin and an iron overload phenotype (based on *in vivo* studies of NCOA4 knockout). Excess free iron is associated with reactive oxygen species generation and oxidative stress and may contribute to neurodegeneration. Further experimentation is necessary to link specific defects in the NCOA4-mediated ferritinophagy pathway to neurodegeneration.

To balance cellular requirements for iron with the potential deleterious effects of free iron, cells possess an intricate system of proteins involved in intracellular iron homeostasis (Pantopoulos et al., 2012). Critically, cellular iron is stored in a non-toxic form in ferritin, which is a cytosolic heteropolymer made of 24 subunits of ferritin heavy and light chains (FTH1/FTL) that can store up to 4500 iron atoms (Arosio et al., 2009). Iron is stored in ferritin during times of iron excess and must be released under periods of iron demand. The predominant pathway for iron release from ferritin is via Nuclear receptor coactivator 4 (NCOA4)-mediated selective autophagy whereby NCOA4 binds ferritin to traffic it to the lysosome where ferritin is degraded and iron is released for use by the cell (Mancias et al., 2014; Figure 1).

Maintaining iron balance in the cell is a complex and highly regulated process, which, if impaired may lead to cell death. Recent studies show that NCOA4 plays a central role in maintaining this delicate balance on a cellular and systemic level (Mancias et al., 2014, 2015; Bellelli et al., 2016). Dysregulation of NCOA4-mediated ferritinophagy disrupts systemic iron homeostasis with deleterious effects on erythropoiesis and regulation of oxidative stress. Furthermore, recent studies show that NCOA4-mediated ferritinophagy modulates susceptibility to ferroptosis (Gao et al., 2016; Hou et al., 2016). As many NDs are associated with defective iron metabolism and autophagy, NCOA4 may play an important role in the onset and development of these disorders. Here, we review the literature to delineate new hypotheses to elucidate the potential significance of NCOA4 in ND.

## NCOA4 Mediates Ferritinophagy and Regulates Intracellular Bioavailable Iron

We recently identified NCOA4 as the selective autophagy receptor mediating delivery of iron-laden ferritin to the lysosome for degradation and release of iron, a process termed ferritinophagy (Mancias et al., 2014). Using a combination of autophagosome enrichment and quantitative mass spectrometry-based proteomics, NCOA4 was identified as one of the most robustly enriched proteins in autophagosomes. Affinity purification-mass spectrometry identified ferritin subunits (FTL, FTH1) and the HERC2 E3 ubiquitin ligase as NCOA4-interacting partners. *In vitro* assays with recombinant FTH1 and NCOA4 demonstrated that NCOA4 binds to ferritin via a direct interaction of a conserved surface arginine (R23) on FTH1 and a C-terminal domain in NCOA4 (only present in NCOA4α) (Mancias et al., 2015). Gryzik et al. (2017) further revealed that FTH1 can bind up to 24 NCOA4 fragments in *in vitro* binding assays. NCOA4 colocalized with ferritin in autophagosomes and lysosomes. Importantly, NCOA4 depletion inhibited delivery of ferritin to the lysosome and thereby ferritin degradation (Mancias et al., 2014) implicating NCOA4 in the selective autophagic degradation of ferritin. The decrease in ferritin turnover due to NCOA4 loss leads to a decrease in bioavailable cellular iron highlighting the importance of NCOA4 in regulating intracellular iron homeostasis.

Flux through the ferritinophagy pathway is determined by NCOA4 levels which are in turn regulated by iron levels in the cell (Mancias et al., 2015). When cellular iron levels are high, NCOA4 interacts with the HERC2 ubiquitin E3 ligase in an iron-dependent manner and is targeted for degradation via the ubiquitin proteasome system. This ensures NCOA4 levels are low under high iron conditions thereby decreasing ferritinophagy and favoring ferritin iron storage. Conversely, under low cellular iron conditions, the NCOA4 and HERC2 interaction is decreased leading to elevated NCOA4 levels and increased ferritinophagy flux to replenish cellular iron levels (Mancias et al., 2015).

The mechanism of NCOA4-ferritin trafficking to the lysosome is unclear with evidence for both classical ATG8-dependent autophagic delivery to the lysosome and a non-classical ESCRT-mediated delivery system involving TAX1BP1, VPS34, ATG9A, and ULK1/2-FIP200 (Mancias et al., 2014; Goodwin et al., 2017). Further research is necessary to understand the mechanism(s) that direct NCOA4 to the lysosome. Alternatively, proteasomal degradation of ferritin has been reported in the context of Ferroportin-mediated iron export (De Domenico et al., 2006). While the multiple reported mechanisms of ferritin degradation may suggest context-dependent modes of ferritin turnover; NCOA4-mediated mechanisms appear to be the predominant means of ferritin degradation.

While NCOA4-mediated ferritinophagy appears to be a conserved function of NCOA4, initial studies of NCOA4 suggested it acts as a nuclear receptor coactivator, albeit a weak one (Alen et al., 1999; Gao et al., 1999; Monaco et al., 2001; Hu et al., 2004; van de Wijngaart et al., 2006). More recently, Lodish and colleagues reported that NCOA4 is stimulated by thyroid hormone promoting its recruitment to chromatin regions associated with transcripts abundant during erythropoiesis (Gao et al., 2017). NCOA4 has also been reported to act as a regulator of DNA replication origin activation via an interaction with the minichromosome maintenance 7 protein (MCM7) (Bellelli et al., 2014). Further work is required to understand the different reported functions of NCOA4 and whether there are context dependent variations in NCOA4 function.

## Physiological Role of NCOA4

Given NCOA4 is a critical component of the intracellular iron homeostasis machinery, understanding its role in modulating systemic iron homeostasis has been of paramount importance. The physiological role of NCOA4-mediated ferritinophagy has been studied *in vivo* initially in the context of erythropoiesis, a highly iron-dependent process. Early studies to understand the relationship between NCOA4 and erythropoiesis showed that NCOA4 mRNA levels were upregulated at erythropoietic sites in zebrafish (Weber et al., 2005). NCOA4 ablation in a zebrafish model leads to defects in globin synthesis and hemoglobinization (Mancias et al., 2015). Dowdle et al. (2014) revealed that NCOA4 is necessary for ferritin turnover and iron homeostasis *in vivo* as NCOA4-deficient mice showed accumulation of ferritin and increased iron deposits associated with splenic macrophages. A systemic NCOA4 knockout mouse model show that loss of NCOA4 leads to accumulation of ferritin in all organs examined. Mice developed iron accumulation in the liver and spleen and signs of iron overload in the serum. While all signs pointed to an iron overload phenotype, these mice developed a mild microcytic hypochromic anemia suggesting an inability to properly utilize the available iron. This anemia was exacerbated by a low iron diet with mice unable to mobilize iron stores from ferritin. An additional study in a systemic NCOA4 knockout mouse model similarly showed anemia in experimental mice that was more pronounced in the immediately post-natal period suggesting a differential temporal dependence of NCOA4 function in erythropoiesis or more generally in iron mobilization from ferritin (Gao et al., 2017). Given the systemic nature of the NCOA4 knockout with associated global dysregulation in iron homeostasis in both of these mouse models, the relative erythroid cell autonomous and non-autonomous contributions toward the anemia phenotype remained unclear until recently. Multiple *ex vivo* cellular models of erythropoiesis suggested a cell autonomous role for NCOA4 in erythropoiesis (Mancias et al., 2015; Ryu et al., 2017). A recent study from our group using a conditional knockout mouse model of NCOA4 has clarified the cell autonomous role for NCOA4 in erythropoiesis (Santana-Codina et al., 2019). *NCOA4^fl/fl^; EpoR-Cre* mice with erythroid specific deletion of NCOA4 developed a pronounced anemia in the postnatal period and a mild hypochromic microcytic anemia at adult stages. Notably, the anemia at both ages in *NCOA4^fl/fl^; EpoR-Cre* mice was less severe than that of mice with systemic NCOA4 knockout suggesting both cell autonomous and non-autonomous roles for NCOA4 in supporting basal erythropoiesis. While these *in vivo* studies did not perform in-depth analysis of the consequences of NCOA4 depletion in the brain, acute systemic depletion of NCOA4 using an inducible *NCOA4^fl/fl^; UBC-cre/ERT2* murine model did reveal accumulation of FTH1 over the time span of a week in the brain suggesting a basal level of dependence on NCOA4 in the brain for FTH1 turnover (Santana-Codina et al., 2019).

An additional finding of interest in NCOA4 null mice was an increase in serum ferritin levels (Bellelli et al., 2016). Ferritin has been reported to be secreted via a non-canonical lysosomal secretory pathway (Cohen et al., 2010). Despite the role of NCOA4 in mediating lysosomal delivery, it appears in NCOA4-deficient mice, ferritin is still secreted via a lysosomal pathway or an alternative one such as the multivesicular body-exosome pathway (Truman-Rosentsvit et al., 2018). This finding of elevated ferritin secretion in the setting of NCOA4 loss was corroborated by a cell culture study showing that loss of NCOA4 accelerated ferritin exocytosis (Kimura et al., 2017). This may have implications in the central nervous system (CNS) where ferritin has been identified in the cerebrospinal fluid and may act as an iron carrier (Todorich et al., 2008).

## NCOA4-Mediated Ferritinophagy and Ferroptosis

In determining the relevance of NCOA4-mediated ferritinophagy beyond erythropoiesis, recent studies have identified a link between NCOA4 and a newly discovered form of iron-dependent cell death, ferroptosis. Ferroptosis is an iron-dependent form of cell death characterized by lipid peroxidation accumulation (Dixon et al., 2012; Stockwell et al., 2017). Ferroptosis can be triggered by multiple mechanisms including iron accumulation leading to reactive oxygen species resulting in lipid peroxidation or via inactivation of the lipid repairing peroxidase, GPx4. Given NCOA4-mediated ferritinophagy modulates intracellular iron levels, ferroptosis sensitivity has recently been shown to be affected by NCOA4 levels (Gao et al., 2016; Hou et al., 2016; Tang et al., 2018). In cell culture models, NCOA4 depletion decreases intracellular bioavailable iron and thereby decreases sensitivity to ferroptosis-inducing insults. On the other hand, NCOA4 over-expression in cellular models increases ferritinophagy flux and thereby increases sensitivity to ferroptosis. Interestingly, in NCOA4 knockout mice, consistent with their iron overload phenotype and elevated basal levels of tissue iron, knockout mice fed an iron-enriched diet developed early liver toxicity and premature death (Bellelli et al., 2016). This was associated with elevated levels of oxidative stress as indicated by increased Gpx and superoxide dismutase (SOD) levels. This suggests that in the context of long-term NCOA4 depletion *in vivo*, sensitivity to ferroptosis may actually be enhanced. This observation may have implications for NDs that have been shown to be associated with defects in autophagy, excess iron accumulation, oxidative stress, and ferroptosis.

## Neurodegenerative Disorders and Iron

While there is no direct evidence for dysregulation of NCOA4-mediated ferritinophagy as a cause for ND, there are multiple lines of correlative evidence related to defective iron metabolism, autophagy, and ferroptosis that suggest a link. Iron is critical for normal physiologic function in the brain with roles in such processes as DNA synthesis, oxygen transport, mitochondrial respiration, and neurotransmitter synthesis (Ward et al., 2014). Iron accumulation is a normal process in the aging brain; however, the mechanism by which this occurs is unknown (for in-depth review of normal iron homeostasis mechanisms in the brain, we refer the reader to: (Ward et al., 2014; Garza-Lombó et al., 2018). Healthy brains accumulate iron in cytosolic ferritin and in iron bound to neuromelanin found in lysosomes (Zecca et al., 2001). Iron normally accumulates in multiple regions of the brain including the globus pallidus, putamen, substantia nigra, caudate nucleus, and red nucleus (Zecca et al., 2004; Angelova and Brown, 2015; Biasiotto et al., 2016). Interestingly, these areas of the brain are the most susceptible to ND. The cells where elevated levels of iron are found are the oligodendrocytes with varying levels found in microglia and smaller amounts of iron in neurons and astrocytes (Rouault, 2013). While iron accumulation is normal with aging, inappropriate iron accumulation and dysregulation of iron homeostasis in combination with alterations in ferritin levels are associated with ND and worse outcomes amongst ND patients. However, it remains unclear whether this is a primary or secondary phenomenon in ND. One potential primary cause of ND related to inappropriate iron accumulation is ferroptotic cellular death as recently demonstrated in models of ND. The first evidence for a link between ND and ferroptosis came from *in vitro* rat organotypic hippocampal slice cultures treated with glutamate to mimic the pathophysiology of stroke and ND (Dixon et al., 2012). Cells were found to undergo ferroptosis that could be prevented by iron chelators and ferrostatin (Dixon et al., 2012). Furthermore, studies in multiple models of ND with genetic (Chen et al., 2015) or pharmacological inhibitors of ferroptosis (Skouta et al., 2014; Do Van et al., 2016) showed that inhibition of ferroptosis decreased neuronal cell death pointing to a potential future therapeutic strategy.

In addition to dysfunction in iron homeostasis as a potential cause of ND, alterations in protein and organelle quality control are associated with ND. While neuronal autophagy activity levels decrease in the normal aging brain, abnormal decreases in autophagy and genetic perturbations in the autophagy-lysosome pathway are associated with ND. Indeed, genetic mouse models of neuronal autophagy deficiency develop ND (Hara et al., 2006; Komatsu et al., 2006). One hypothesis links the changes in iron homeostasis seen in both normal aging and ND with decreased levels of autophagy. In support of this, there are a number of ND diseases with genetic alterations in autophagy machinery that are associated with inappropriate iron accumulation (for an exhaustive review, see Biasiotto et al., 2016). Direct links between defective autophagy, iron accumulation, and ND are illustrated by a newly identified form of Neurodegeneration with Brain Iron Accumulation (NBIA: a group of genetic neurological disorders with abnormal accumulation of iron in the basal ganglia), static encephalopathy of childhood with neurodegeneration in adulthood (SENDA), due to a mutation in WDR45 (Saitsu et al., 2013). WDR45 encodes WD-repeat domain 45 protein, also known as WIPI4, which has a role in autophagy initiation. Pathogenic mutations in WDR45 seen in SENDA patients lead to cellular defects in autophagy, diminished lysosomal function, iron accumulation, and oxidative stress (Seibler et al., 2018). Accordingly, patients presenting with these deleterious mutations have been found to have large iron depositions in the globus pallidus and substantia nigra (Saitsu et al., 2013). Interestingly, in *ex vivo* models of WDR45 loss of function, pharmacologic stimulation of the autophagy pathway with Torin1 leads to normalization of iron levels (Seibler et al., 2018). The precise molecular mechanistic details linking alterations in autophagy function to changes in iron levels have not been determined in this disease or in general in ND. However, given NCOA4-mediated ferritinophagy links iron homeostasis and autophagy, it is tempting to hypothesize that defects in ferritinophagy may underlie inappropriate accumulation of iron in ND. We next review the role of altered iron metabolism and autophagy in the two main neurodegenerative disorders, Alzheimer’s Disease and Parkinson’s Disease.

Alzheimer’s disease is the most common cause of dementia or cognitive impairment in patients older than 65 years old and is the sixth leading cause of death in the United States (Atri, 2019). AD is characterized by accumulation of intracellular and extracellular aggregates of β-amyloid (Aβ) as well as tau protein leading to neuronal dysfunction and neurodegeneration. Interestingly, high concentrations of redox-active metals, including iron, are present in these aggregates. Excess iron in these aggregates may lead to deleterious iron loss in neurons affecting neuronal function (Roberts et al., 2012). Furthermore, altered iron homeostasis has been linked to the initial misfolding of tau and β-amyloid. Specifically, amyloid precursor protein (APP) has a putative iron response element (IRE) in its 5^′^ untranslated region under the control of the IRP1/2 post-transcriptional regulatory system (Rogers et al., 2002). Iron excess may decrease IRP1/2 binding on this IRE thereby increasing APP translation making it available for β-amyloid production. A hallmark of AD is oxidative stress-induced damage stemming from a number of factors but likely accelerated by inappropriate accumulation of redox-active metals, including iron. Indeed, reducing levels of iron/redox-active metals has recently become a clinical target in neurodegenerative diseases in general and in AD specifically (Nuñez and Chana-Cuevas, 2018). Recent phase II clinical trials of chelation therapy for AD patients revealed promising stabilization or improvement in AD symptoms and decreases in Aβ in plasma or cerebrospinal fluid (Ritchie et al., 2003; Lannfelt et al., 2008). While inappropriate accumulation of iron is clearly associated with AD in animal models of the disease and in human patients as detected by MRI (Schenck, 2003), the relationship between altered iron homeostasis and the pathogenesis of AD is unclear. Whether increased iron is a cause or a secondary effect of the pathogenic amyloid aggregates remains unclear.

Parkinson’s disease is the second most common ND and is characterized by selective loss of dopaminergic neurons in the substantia nigra initially leading to motor dysfunction. Similar to AD, PD is also characterized by accumulation of aggregated alpha-synuclein in Lewy bodies, mitochondrial dysfunction, and oxidative stress (Ward et al., 2014). PD patients also demonstrate increases in total iron concentration in the substantia nigra. For instance, increased DMT1 expression (Salazar et al., 2008) and activating mutations in Transferrin both lead to an increase in cytosolic iron and are correlated with worse outcomes for PD patients (Rhodes et al., 2014). Interestingly, an increase in redox-active iron may also be related to a decrease in ferritin synthesis in PD patients (Faucheux et al., 2002). In general PD patients with the most severe neuronal loss have the highest levels of redox-active iron (Faucheux et al., 2003). As redox-active iron is clearly associated with generation of reactive oxygen species, inappropriate iron accumulation may be central to PD pathogenesis. However, as for AD, it is unclear whether this is a cause of a symptom of PD pathogenesis.

NCOA4 expression and function in the brain and its role in ND is an unexplored field of study. There is evidence of NCOA4 expression in the murine and rat brain at both mRNA and protein levels (Kollara and Brown, 2010). However, there are no studies of NCOA4 expression levels or function during aging or in pathological specimens from ND patients. Furthermore, there are no known pathogenic mutations in NCOA4 associated with ND. Presumably, with a decrease in global autophagy as is observed in some ND, ferritinophagy flux would also be decreased (Figure 1). As described above, the outcome of decreased NCOA4 levels and ferritinophagy flux as it relates to iron status in the cell varies depending on the system examined. *In vitro* cellular models of NCOA4 depletion show a decrease in bioavailable iron due to a decrease in ferritin degradation resulting in trapped iron (Mancias et al., 2014). This leads to decreases in bioavailable iron, lower levels of oxidative stress, and decreased sensitivity to ferroptosis. This would suggest that NCOA4 depletion in the brain in the context of ND could be protective. However, in the setting of constitutive systemic NCOA4 depletion in animal models, animals show signs of iron overload and increased sensitivity to oxidative stress (Bellelli et al., 2016). These discordant results may be a consequence of a number of differences between *in vitro* versus *in vivo* systems, including the time scale of NCOA4 loss, relative dependence on NCOA4-mediated ferritinophagy for iron balance in cell culture models (most of which are tumor-derived cell lines) versus normal tissues, and differences in the iron availability between *in vivo* growth conditions and cell culture models which are predominantly grown in transferrin-rich serum-containing media. The results from *in vivo* models of NCOA4 depletion showing iron overload and increased sensitivity to oxidative stress could align with prior findings of decreased autophagy, increased iron accumulation, and oxidative stress demonstrated in ND (Figure 1). However, additional studies are required to assess the expression, regulation, and function of NCOA4 in the brain under physiologic and ND conditions. We speculate that long-term NCOA4 depletion in the brain in the context of animal models of ND would worsen the ND phenotype due to a further inappropriate accumulation of free iron and resulting oxidative stress as has been previously demonstrated in systemic long-term depletion of NCOA4 (Bellelli et al., 2016). Newly available conditional NCOA4 knockout models with targeted deletion of NCOA4 in the brain will be instrumental in understanding the potential role of NCOA4 function in the brain and in ND specifically.

## Author Contributions

MQ and JDM performed the literature searches and wrote and edited the manuscript.

## Conflict of Interest Statement

JDM is an inventor on a patent pertaining to the autophagic control of iron metabolism. The remaining author declares that the research was conducted in the absence of any commercial or financial relationships that could be construed as a potential conflict of interest.
